# Eosinophilic Cystitis with Eosinophilic Cholecystitis: A Rare Association

**DOI:** 10.1155/2013/146020

**Published:** 2013-10-01

**Authors:** F. Mallat, W. Hmida, S. Mestiri, S. Ziadi, B. Sriha, M. Mokni, F. Mosbah

**Affiliations:** ^1^Urology Department, Sahloul Hospital, Sousse, Tunisia; ^2^Pathology Department, Farhat Hached Hospital, Sousse, Tunisia

## Abstract

We describe a rare case of eosinophilic cystitis associated with eosinophilic cholecystitis in a 30-year-old patient who underwent bladder biopsy for irritative voiding symptoms and routine elective cholecystectomy for gallstones. Diagnosis was confirmed by histopathological examination. The rarity of this condition prompted us to report this entity in which no specific cause could be found.

## 1. Introduction

Eosinophilic cystitis is a rare form of bladder inflammation. It is a poorly understood clinicopathological condition first described in 1960 [1, 2]. It usually causes irritative voiding symptoms and can sometimes simulate a malignant lesion.

Eosinophilic cystitis can be associated with eosinophilic infiltration of other organs, such as the gastrointestinal tract and liver [3, 4]. To our knowledge, association of eosinophilic cystitis with eosinophilic cholecystitis has never been reported in the literature.

We report a case of eosinophilic cystitis associated with eosinophilic cholecystitis in which no specific cause could be found.

## 2. Case Report

A 30-year-old patient presented with difficulty in voiding, slow stream of urine, an elevated urinary frequency, suprapubic pain, and enuresis for 2 years with gross haematuria in the last 2 months. The patient also complained for few months of intermittent abdominal pain and tenderness in the right upper quadrant. The patient had no significant personal or family history of allergic disorders.

At physical examination, the patient was afebrile, and there was no icterus, cyanosis, or pallor. Murphy's sign was positive.

**Figure 1:**
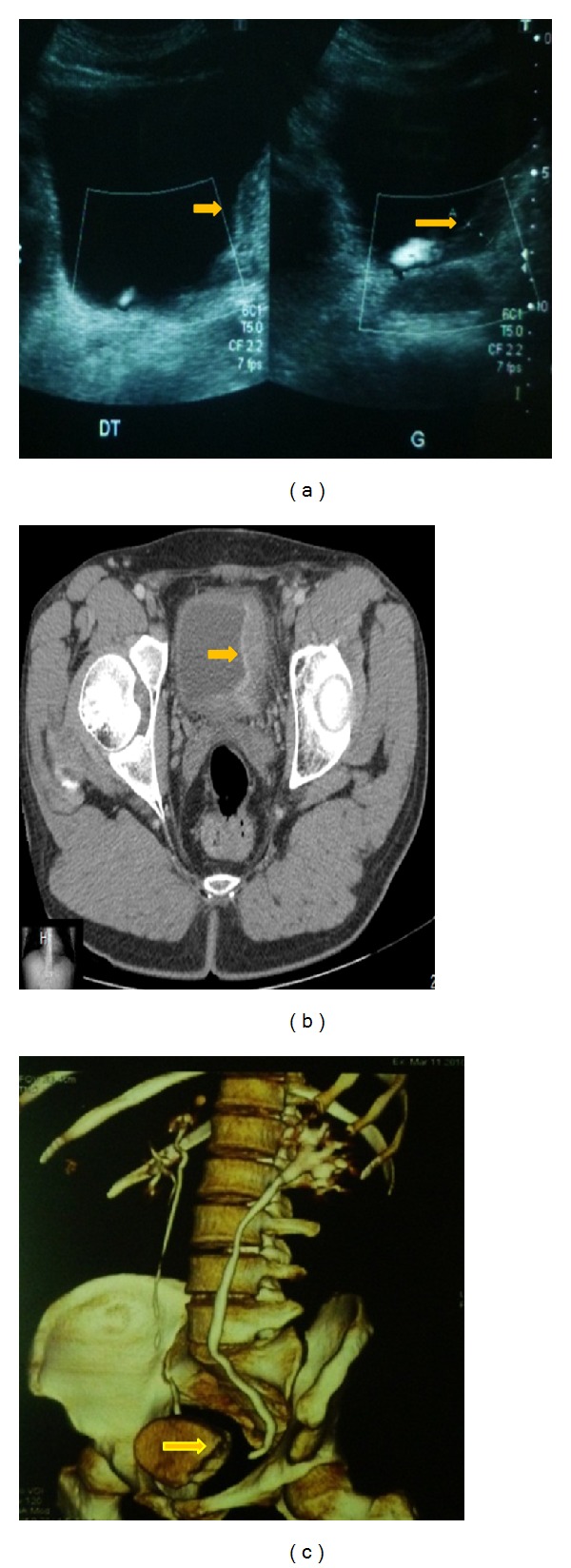
(a) Ultrasound: large mass of the left wall of the urinary bladder. ((b) and (c)) Contrast-enhanced CT scan shows marked and diffuse bladder wall thickening, bladder tumour mainly on the left but extending to the midline anteriorly and beyond the midline posteriorly with moderate dilatation of the upper ureter.

An abdominopelvic ultrasound revealed an echogenic and diffuse wall thickening of the left side of the urinary bladder (Figure 1(a)) with moderate dilatation of the upper urinary tract. There was a retracted gallbladder with calculi and circumferential thickening of the wall, exceeding 9 mm (Figure 3(a)). Liver, common bile duct, and the rest of the abdominal viscera were unremarkable.

CT scan showed a bladder tumour mainly located on the left side but extending to the midline anteriorly and beyond the midline posteriorly (Figures 1(b) and 1(c)) with an extraparietal extension. Moderate dilatation of the left upper urinary tract was found. Enlarged left-sided lymph nodes were also noticed. The gallbladder was retracted with microlithiasis and diffuse thickening of its wall (Figure 3(b)).

A diagnosis of both urinary bladder tumor and chronic cholecystitis with cholelithiasis was made.

Laboratory tests on admission revealed a WBC count of 8600/mm^3^ with an eosinophil count of 33% (normal 0%–4%). Serum IgE level was 550 U/L (normal < 140 U/L). The serum amylase level was 94 U/L (normal 25–115 U/L) and serum lipase was 215 U/L (normal 114–286 U/L). RAST testing for a battery of allergens, including common foods, was negative. Other laboratory tests, including liver function tests, serum lipid profile, and serum immunoglobulines, were normal. Stool studies for ova and parasites were negative.

Direct examination of the urine confirmed gross haematuria, but no microorganisms were seen. Urine cytology showed no malignant cells. Urine cultures were negative.

**Figure 2:**
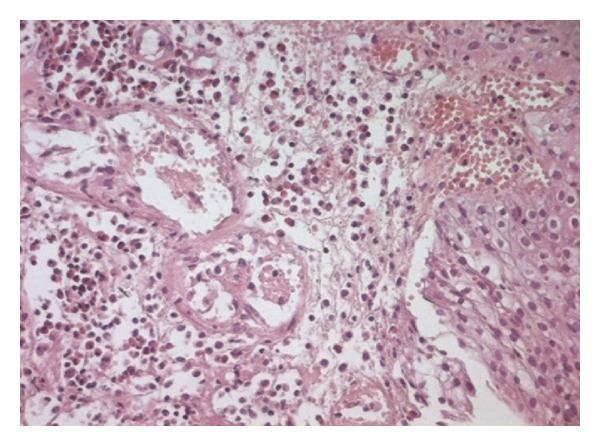
H.E. ×250 vascular congestion of the urothelial mucosae with an inflammatory infiltrate consisting predominantly of eosinophils, suggesting eosinophilic cystitis.

**Figure 3:**
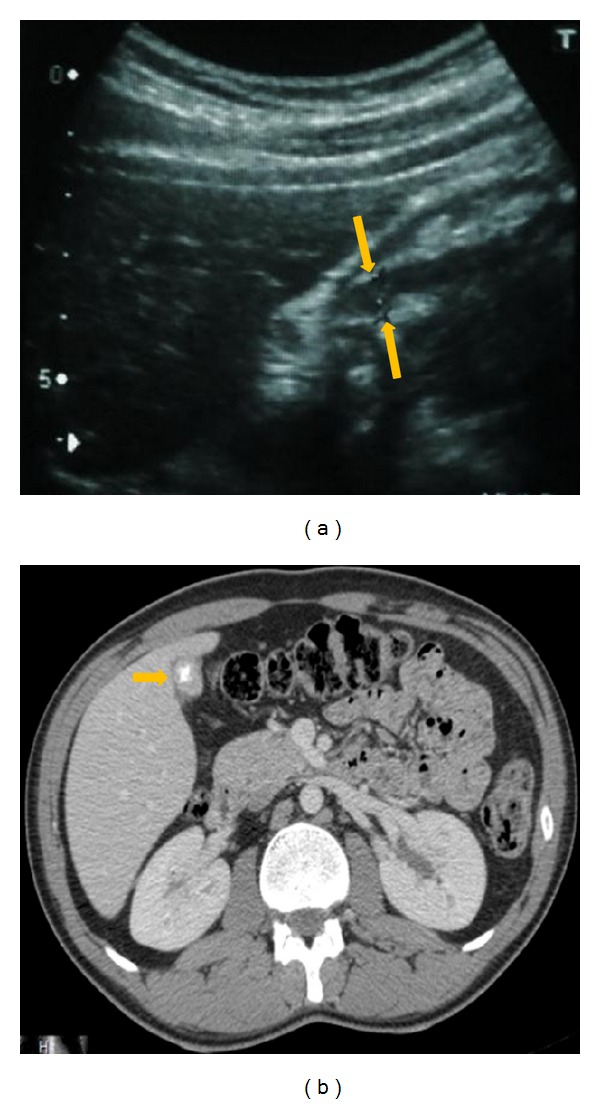
Ultrasound (a) and CT scan (b) showing the gallbladder was retracted with microlithiasis and diffuse and circumferential thickening of its wall.

At cystoscopy, a large papillary and solid lesion was seen on the left side of the bladder. Random bladder biopsy revealed focal erosion of the mucosa, edema, vascular congestion, and a dense polymorphous infiltrate made of few lymphocytes and numerous eosinophils (Figure 2). Diagnosis of eosinophilic cystitis was made.

A medical treatment was indicated with Prednisone 20 mg/day and sodium diclofenac 50 mg every 8 hours, with a good response. At cystoscopy 8 weeks later, the bladder appeared normal. The CT scan changes had completely resolved. The eosinophil count normalized.

Unfortunately, symptoms recurred three months later on tapering down the steroids. The patient responded well to a 6-week course of Prednisone 40 mg/d, and sodium cromoglycate was added to the patient's treatment and helped in tapering down steroids to a maintenance dose of 10 mg/d. After 9 months, treatment was stopped, and the patient was doing well. A control cystoscopy was performed, without significant findings. A control biopsy revealed a nonspecific inflammation.

The patient underwent this year a laparoscopic cholecystectomy under general anesthesia. Peroperatively, a thick-walled retracted gallbladder was noticed.

At gross examination, the gallbladder measured 5 cm in length. Cut section showed a thickened wall and calculi in the lumen.

**Figure 4:**
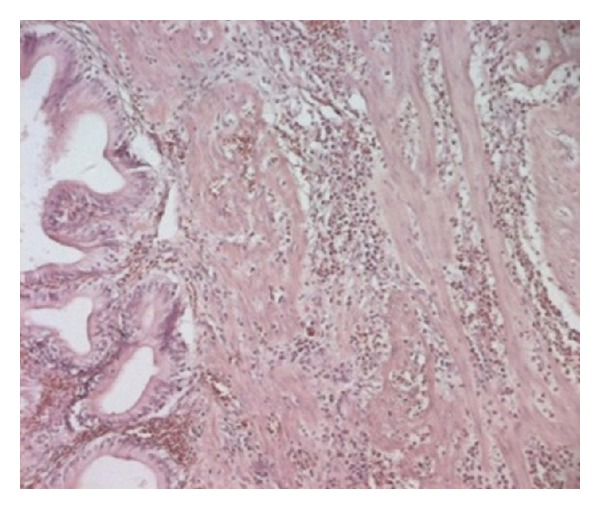
Inflammatory infiltrate consisted predominantly of eosinophils, suggesting eosinophilic cholecystitis.

Histologically, the mucosae were lined by a regular columnar epithelium and all the layers of gallbladder were heavily infiltrated by eosinophils, associated with oedema and vascular congestion (Figure 4). The adventice showed both fibrosis and thickened wall arterioles. All these features were concordant with eosinophilic cholecystitis with cholelithiasis.

Postoperatively, the patient had an uneventful recovery and was discharged on oral analgesics. Control computed pelvi-abdominal tomography was normal.

## 3. Discussion

Eosinophilic cystitis is an infrequent and poorly understood inflammatory condition of the urinary bladder. The etiology of eosinophilic cystitis is still obscure.

In reported cases, eosinophilic cystitis is preceded or is synchronous with development of other gastrointestinal or liver lesions in only 4.5% of cases [5]. To our knowledge, eosinophilic cystitis that is associated with eosinophilic cholecystitis has never been reported in the literature.

This association has neither any specific clinical manifestation nor laboratory features and cannot be clinically distinguished from ordinary cystitis and cholecystitis. Imaging studies of patients with eosinophilic cystitis are not specific. It can show thickening of the wall and sometimes mimic a neoplastic process [6, 7].

The gold standard for diagnosing these lesions is histopathological examination, showing an infiltration of the wall by an inflammatory infiltrate, made predominantly of eosinophils.

In eosinophilic cystitis, peripheral blood eosinophilia can be found in 43% of cases [8] and up to 50% of patients with a history of allergy or atopy. Our patient had peripheral blood eosinophilia with no history of allergy or atopy.

Eosinophilic cholecystitis represents 0.25 to 6.4% of all cholecystitis [9, 10]. Usually, it is an acalculous cholecystitis, but in 40% of cases, calculi are found [11].

Eosinophilic cholecystitis has been reported alone or in combination with eosinophilic gastroenteritis, eosinophilia-myalgia syndrome, idiopathic hypereosinophilic syndrome, parasitic infestations (Clonorchis sinensis and hydatid cyst disease) and antibiotics (erythromycin and cephalosporins) [12, 13].

A suspicion of this entity can be kept if peripheral eosinophilia is present.

In our case, the hypothesis of eosinophilic cholecystitis was raised because of both peripheral eosinophilia and histopathologic confirmation of eosinophilic cystitis and was confirmed by histopathological examination of cholecystectomy specimen.

Cholecystectomy appears to be the adequate treatment for eosinophilic cholecystitis. Nevertheless, in two cases of eosinophilic cholecystitis with eosinophilic cholangitis, one patient was cured with steroids and the second was treated with antibiotics [14]. Treatment of eosinophilic cystitis is not standardised. It usually begins by a removal of the antigen, if present. Initial medical treatment should include antihistaminics and nonsteroidal anti-inflammatory drugs. It is recommended to start with hydroxyzine 20 mg every 8 hours. If somnolence occurs, the patient should be changed to another antihistaminic (Cetirizine). In refractory or severe cases presenting with ureteral infiltration, corticosteroids should be added [15]. The success rate of this therapy reaches 80% and 100% when corticosteroids are added [15].

In adults, eosinophilic cystitis usually has a chronic course with periodic recurrences [16], as seen in our patient. Around 7% of cases show an aggressive course with recurrent haematuria, impaired renal function, and resistance to medical treatment [17]. In these cases, nephroureterectomies, partial or total cystectomy, and augmentation cystoplasties can be performed [17].

## 4. Conclusion

In our case, the presence of peripheral blood eosinophilia and the double localization of the disease suggests that eosinophilic infiltration of the gallbladder and the urinary bladder wall is rather a generalised reaction.

If a patient presents only with symptoms of cholecystitis, and a postoperative histopathological diagnosis of eosinophilic cholecystitis is made, the patient must be investigated to rule out other associated disease conditions, which may have a worse prognosis than cholecystitis itself.
